# Capabilities of Single Cell ICP-MS for the Analysis of Cell Suspensions from Solid Tissues

**DOI:** 10.3390/nano13010012

**Published:** 2022-12-20

**Authors:** Roberto Álvarez-Fernández García, Lucía Gutiérrez Romero, Jörg Bettmer, Maria Montes-Bayón

**Affiliations:** 1 Department of Analytical Chemistry, Faculty of Chemical Sciences, Complutense University of Madrid, 28040 Madrid, Spain; 2 Instituto de Investigación Sanitaria del Principado de Asturias (ISPA), Department of Physical and Analytical Chemistry, Faculty of Chemistry, University of Oviedo, Julian Clavería 8, 33006 Oviedo, Spain

**Keywords:** disaggregated tissues, single cell ICP-MS, spleen, liver, HepG2

## Abstract

Single cell elemental (SC) analysis of isogenic cell cultures can be done using inductively coupled plasma (ICP-MS) detection. However, 2D cell cultures are just models to simplify the complexity of real tissue samples. Here, we show for the first time the capabilities of the technique (SC-ICP-MS) to analyze single cell suspensions of isolated cells from tissues. An optimized cocktail of proteolytic and collagenolytic enzymes was applied in a single preparation step with cellular yields up to 28% using 0.5 g of fresh rat spleen and liver, respectively. The retrieved cells revealed adequate morphology and stability to be examined by SC-ICP-MS. Quantitative elemental analysis of P, S, Cu, and Fe from disaggregated cells from rat spleen and liver tissues revealed levels of Fe of 7–16 fg/cell in the spleen and 8–12 fg/cell in the liver, while Cu was about 3–5 fg/cell in the spleen and 1.5–2.5 fg/cell in the liver. Evaluation of the transmembrane protein transferrin receptor 1 (TfR1) expression levels in disaggregated cells was also conducted by using a Nd-labelled antibody against this cell surface biomarker. Quantitative results showed significantly lower expression in the disaggregated cells than in the cell model HepG2, in agreement with the overexpression of this biomarker in tumor cells. In this proof of concept study, the tissue disaggregation protocol has shown to maintain the elemental intracellular content of cells as well as the presence of relevant antigens. This opens a completely new area of research for SC-ICP-MS in tissue samples as a complementary strategy with validation capabilities.

## 1. Introduction

Heterogeneity between individual cells is obvious in tissues and organisms, but it can also be observed in populations of isogenic cells that have been cultured under identical conditions [1]. In fact, the cell to cell variation of the genes, transcripts, and proteins can be the cause of several diseases, including cancer, as well as neurological and developmental disorders [2]. Similarly, an alteration of metal control which is tightly regulated by cells (homeostasis) can be associated with the initiation of important pathological conditions such as diabetes or cancer. Therefore, the analysis of cellular components and metabolism at the single cell level is not only an important way to reveal the heterogeneity and differentiation of cells, but also an effective strategy to accurately investigate the relationship between the contents of elements or biomolecules and related signaling pathways. In this regard, several authors have investigated the heterogeneity in the metal composition of cells using cultured models [3,4]. This scenario could serve as a first approximation to the real situation in tissues or living organisms. However, the analysis of the elemental composition in individual cells from tissue samples is still challenging.

Most authors have sought to use inductively coupled plasma-mass spectrometry (ICP-MS) connected on-line to laser ablation (LA-ICP-MS). This strategy is limited by several drawbacks [5]. Regarding the sample preparation, the tissues are generally embedded in paraffin or plastic molds to obtain a thickness of less than 10 µm, so that single cells might already become visible. In any case, such sample treatment might compromise the stability of the target elements within the cell compartments and the sample thickness might result in the ablation of multiple cell layers [6]. In addition, to achieve cellular and subcellular resolution, one of the main limitations is the diameter of the laser beam or spot size (considering cell diameters ranging 10 to 20 µm). Recently, by manipulating the optical system, the reduction of the spot size to 1 µm or even lower [7,8] has been possible. On the other hand, the development of low-dispersion laser ablation chambers has contributed to the increased lateral resolution. The wash-out times of these chambers (1–5 ms) [9] have been reduced from conventional ones (0.5–30 s), so that the sample aerosol is extracted more efficiently towards the plasma reducing aerosol dispersion. Perhaps the most successful combination for single cell analysis in tissue samples has been the use of mass cytometry coupled to a laser ablation system [10,11]. Single cell mass cytometry allows the simultaneous analysis of ~40 cellular markers by previous labelling using lanthanide-containing antibodies. Imaging mass cytometry uses a high-resolution laser coupled to the mass cytometer for the successive ablations of small portions of tissue (~1 μm^2^) [12].

However, the analysis of cell suspensions from disaggregated tissues has been often the way to conduct flow cytometry in order to compare the results of immunohistochemistry from tissue samples. Thus, one way to validate the results that are obtained by the previously described elemental imaging techniques of single cells from tissue would be the disaggregation of the material to obtain single cell suspensions and their introduction into ICP-MS using pneumatic nebulization systems. In this case, the previously mentioned risks that are associated with laser-based approaches, particularly regarding specimen preparation, are negligible. Tissues are complex matrices in which cells are embedded in an extracellular matrix, within which neighboring cells are anchored to each other via cell–cell junctions on the cell membrane. Therefore, several enzymes are necessary to break the extracellular matrix (e.g., collagenase or hyaluronidase), cleave the cell–cell junctions (e.g., trypsin or papain), and prevent cell aggregation (e.g., DNAase-I) [13]. Finally, the cells need to be intact for further ICP-MS analysis. 

In this work, we propose the combination of a sample preparation workflow to obtain single cell information from disaggregated tissue samples and a pneumatic sample introduction system for single cell ICP-MS. As proof of concept, the evaluation of the analytical workflow, from fresh tissues to single cell ICP-MS analysis will be carried out for constitutive elements (e.g., S, P, Fe, Cu) using liver and spleen tissue samples from control rats as well as cell cultures of the HepG2 cell line (human liver cancer) for comparison. In addition, the possibility of applying such preparation workflow for the quantitative analysis of protein markers (e.g., transferrin receptor 1, TfR1) using a labelled antibody will be also evaluated for the first time. 

## 2. Materials and Methods

### 2.1. Reagents and Materials

All solutions were prepared using 18.2 MΩ cm ultrapure water that was obtained from a PURELAB Flex 3 system (ELGA VEOLIA, Wycombe, UK). For tissue dissection, disposable sterile scalpels (Swann-Morton, Sheffield, UK) were used. After dissection, minced tissues were digested by using accutase (Gibco, Life Technologies, Madrid, Spain). FalconTM Nylon cell strainers of 40 µm purchased from Fisher Scientific (Madrid, Spain) were used to filter the resulting cells and avoid the presence of undigested tissue. 

For cell fixation, a buffered aqueous solution of formaldehyde 4% (*v*/*v*) (VWR Chemicals, Radnor, PA, USA) was used. Phosphate buffered saline (PBS), tris buffered saline (TBS), and bovine serum albumin were obtained from Sigma Aldrich (St. Louis, MO, USA, EE. UU.). Mouse antihuman TfR1 monoclonal antibody was purchased from R&D Systems (Minneapolis, MN, USA). The antibody was labelled with 142Nd using a Maxpar X8 antibody labelling kit (Fluidigm, San Francisco, CA, USA) following the manufacturer instructions [14]. For the partial reduction of the antibody, tris(2-carboxyethyl)phosphine (TCEP) from Sigma Aldrich was used. Centrifugal filter units of 3 kDa and 50 kDa (Amicon Ultra 0.5 mL, Merck Millipore, Darmstadt, Germany) were used for the subsequent purification steps. 

For the quantification in SC-ICP-MS analysis, external calibrations were carried out with P, S, Cu, Fe, and Nd ICP standards from CertiPUR^®^ (1000 mg L^−1^) that were purchased from Merck (Darmstadt, Germany). In this context, the transport efficiency of the liquid standards was determined using a 30 nm colloidal gold nanoparticle standard (LGCQC5050) that was produced by LGC (Teddington, UK) with a known particle number concentration and following the particle frequency method [15].

### 2.2. Cell Cultures

The human hepatocarcinoma cell line HepG2 was obtained from the Biotechnological and Biomedical Assays Unit of the Scientific and Technical Services (SCTs) at the University of Oviedo. The cells were grown in T-25 flasks with Dulbecco’s modified eagle medium (DMEM, Labclinics, Barcelona, Spain) supplemented with 10% fetal bovine serum (Gibco) and 5 µg mL^−1^ plasmocin prophylactic (InvivoGen, Nucliber, Madrid, Spain) at 37 °C in a 5% CO_2_ atmosphere. For the SC-ICP-MS experiments, the cells were washed with TBS three times and collected by trypsinization. 

### 2.3. Animal Models

Liver and spleen tissue samples were obtained from control Wistar rats and provided by the Animal Testing Unit Laboratory of the University of Oviedo. All tissue samples were obtained from animals that were sacrificed for other experiments or practical courses within the University of Oviedo. Therefore, no animals were sacrificed exclusively for this work. The animals were controlled and kept in constant environmental conditions and all animal research protocols were carried out in accordance with the institutional guidelines of the University of Oviedo and approved by the Animal Research Ethical Committee of the University of Oviedo (ref. PROAE 34/2019) and by the Institutional Ethics Committee of the Principado de Asturias (ref. 255/19). 

### 2.4. Treatment with Accutase

To isolate single cells from tissues, portions of about 0.5 g of rat spleen and liver were placed in a 15 mL tube and washed three times by centrifugation (5 min × 100 g) with 5 mL of TBS. After each centrifugation cycle, the supernatant was discarded to clean any remaining blood or other unwanted biological material. Then, the tissues were minced into small pieces with a scalpel to increase the total surface area of the tissue and washed again with TBS. The minced tissue was covered with Accutase (Gibco). Accutase is an enzymatic cocktail with proteolytic, collagenolytic, and DNase activity, which has presented higher total cell yield compared with using similar enzymes [16,17]. As mentioned before, this combination of enzymatic activities allows for the degradation of the extracellular matrix of the tissue, cleave cell–cell junctions, and prevents cell aggregation in the resulting cell suspension. 

Different incubation times were assayed from 60 min to 180 min. After the corresponding incubation time at room temperature with orbital shaking, the suspensions were filtered by using cell strainers of 40 µm to avoid the presence of undigested tissue or aggregates from the cell suspension. 

### 2.5. Single Cell ICP-MS and Additional Instrumentation

All the single cell measurements were performed on the iCAPTM TQ ICP-MS (ThermoFisher Scientific, Bremen, Germany). The instrument was equipped with the Single Cell Sample Introduction System for ICP-MS (SC-SIS, Glass Expansion, Weilburg, Germany). All the connections are used as provided by the manufacturer. It contains a high-efficiency concentric glass nebulizer with a sample flow of 10 µL/min and a low volume spray chamber made of borosilicate glass. The system includes a make-up gas inserted into the spray chamber through the use of an inert microJet adapter with 0.01 to 1 L/min flow rate range to avoid deposition of cells into the walls of the spray chamber. Sample interface includes a zero dead volume PEEK nebulizer connector also provided by the manufacturer. A diagram of the commercial system is included in Figure S1. Washing solutions, calibration standards, and cell suspensions were pumped to the ICP-MS at a low flow rate of 10 µL min^−1^ using the syringe pump SP101i (World Precision Instruments, Sarasota, FL, USA) fitted with a 1 mL Hamilton syringe (Hamilton, Reno, NV, USA). Different measurement modes (single quadrupole SQ or triple quadrupole TQ) and collision/reaction gases were used for the measurement of each isotope: ^31^P (TQ-O_2_: ^31^P^+^ > ^31^P^16^O^+^), ^32^S (TQ-O_2_: ^32^S^+^ > ^32^S^16^O^+^), ^56^Fe (SQ-H_2_), ^63^Cu (SQ-He), ^142^Nd, and ^197^Au (SQ). The operation conditions of the iCAP TQ ICP-MS are summarized in Table S1.

After tissue digestion, the obtained cell suspensions were counted precisely by flow cytometry (Cytoflex S from Beckman Coulter, Brea, CA, USA), and the presence of cells and the absence of cell debris or cell aggregates was investigated. At this point, the obtained cells were also observed under an optical microscope (Nikon TMS Inverted microscope, Tokyo, Japan). 

### 2.6. Cell Fixation and Labelling with TfR1 Antibody

After tissue digestion or cell detachment, aliquots of 10^6^ cells of the liver, spleen, and HepG2 cells were prepared. These cells were fixed to prevent cell degradation during the washing and labelling steps by suspending the cell pellet in 500 µL of 4% buffered formaldehyde and incubating for 15 min at room temperature. After fixation, cells were washed by centrifugation (500 g × 5 min) using a 3% BSA in TBS solution. Finally, the cells were labelled with the ^142^Nd-conjugated antibody as previously optimized [18]. Briefly, the washed cell pellet was resuspended in 200 µL of the antibody solution in 3% BSA in PBS. The cells were incubated with the antibody for 30 min at room temperature. After cell tagging, the cells were washed three more times with 500 uL of TBS (up to a total volume of 1500 uL) to reduce the background of Nd and all constitutive elements, especially phosphorus, and re-suspended in 500 uL of ultrapure water for SC-ICP-MS analysis.”

### 2.7. Data Treatment

For the data treatment of the single cell ICP-MS experiments, an iterative procedure that was previously established was followed [19]. In this work, cell events were considered by selecting all the data points where the intensity was four times the standard deviation (4 σ) above the mean of the background signal. The mass of the investigated elements in each individual cell was then calculated by external calibration and considering the mean intensity of the elemental standards, their transport efficiency, the sample flow rate, and the dwell time, as previously described [19].

## 3. Results and Discussion

### 3.1. Optimization of the Disaggregation Treatment with Accutase^TM^

**Figure 1:**
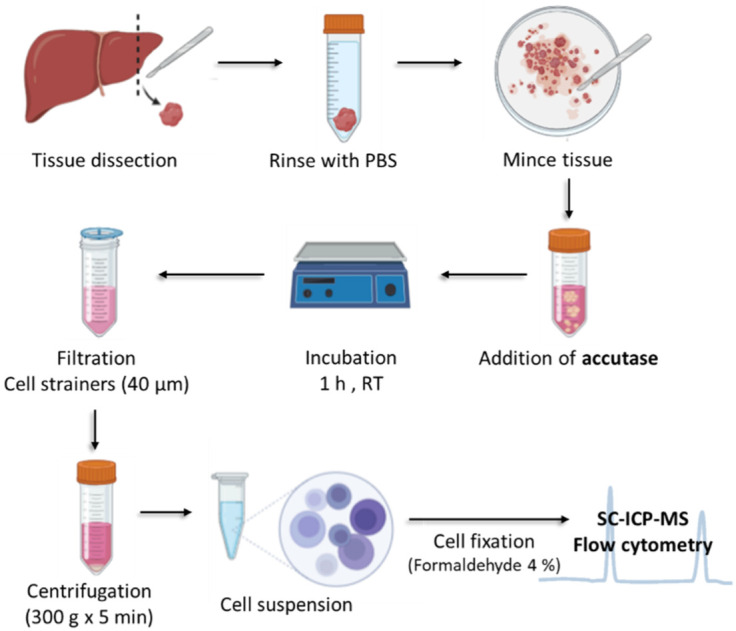
Workflow to prepare a single cell suspension from a solid tissue.

The treatment with ACCUTASE™, a cell detachment solution containing proteolytic and collagenolytic activities could serve as an interesting possibility for solubilizing the tissues and extract intact cells. For this aim, fresh tissues of liver and spleen from rats were first minced to obtain small pieces of about 0.5 cm^2^ (see Figure 1) and thoroughly washed to eliminate the remaining red blood cells.

Furthermore, the pieces were treated with Accutase™ as described in the procedures section and incubated at room temperature during different incubation times: 60, 120, and 180 min for optimization of the process using fresh liver samples. The incubation time in the digestion cocktail seems to play a role in cell viability and aggregation. Incomplete digestion can result in an increase in cell aggregates and, on the other hand, excessive digestion can provide significantly decreased cell viability as well as increased cell debris and fragments in the suspension. It is, therefore, critical to determine the correct incubation time for the specific digestive enzymes and tissues that are involved in each experiment. Afterwards, the suspension should be filtered through 40 µm filtration devices and finally suspended to conduct further fixation (see Figure 1).

**Figure 2:**
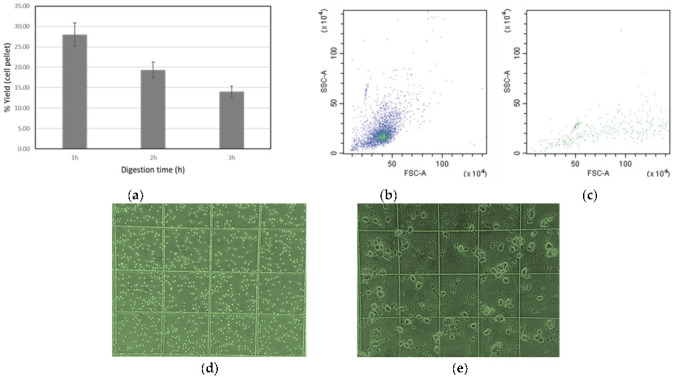
(**a**) Recovery of the disaggregation process using Accutase in liver tissues, calculated as pellet weight with respect to the tissue weight; (**b**) flow cytometry analysis of the cells that were derived from the spleen and (**c**) from liver. Micrographs of the cells that were obtained after enzymatic digestion from rat (**d**) spleen and (**e**) liver tissues (0.25 mm × 0.25 mm grid size).

Figure 2a shows that there were significant differences between 60 and 120 min incubation times with the enzymatic cocktail and the best results were obtained using 60 min achieving up to 28% (*w*/*w*) cell extraction efficiency. Longer times revealed the presence of significantly higher cell debris and lower recoveries. Therefore, 60 min was used as the final incubation time for both the spleen and liver tissues. 

To further prove the integrity of the extracted cells, flow cytometry was used. In this case, the passing of cells one by one through a laser beam causes a scattering of the light both frontal and lateral that can be registered in two separated detectors. The forward scatter light is proportional to the cell size. The side scatter light informs about the internal complexity or granularity of the cell. After enzymatic digestion and filtration of the liver and spleen tissues, the fixed and washed cells were suspended in 500 µL of TBS. At this point, both cell suspensions were injected into the flow cytometer at the same dilution factor registering both side scatter (SSC) and forward scatter (FSC) light from the blue laser (488 nm). The obtained results are shown in Figure 2b,c. Especially for the spleen sample (Figure 2b), a homogeneous cell population was observed in which the fraction of cell aggregates or cell debris was negligible. Such data constitute evidence of the maintenance of the cell integrity during the tissue enzymatic digestion, which is critical for further analysis of intracellular components by SC-ICP-MS. Out of these results, it is also possible to observe that the disaggregation of the liver samples generates a lower cell density in comparison to the spleen. Since flow cytometry allows absolute cell counting considering the sample flow rate (60 µL min^−1^) and the acquisition time (3 min), quantitative numbers were obtained. Taking into account the dilution of the sample, a cell number concentration of 1.9–2.5 *×* 10^6^ and 4–6 × 10^5^ cell mL^−1^ were obtained for the initial cell suspensions of the spleen and liver samples, respectively. Considering that the disaggregation yield was similar for both tissues, it is possible to conclude that the spleen cells should be significantly smaller than those that were obtained from the liver. In fact, if a closer look is taken at the FSC (cell size), the flow cytometry results also confirm the presence of larger cells in the liver sample [20].

The obtained results by flow cytometry were confirmed by microscopy observations. The obtained cell suspensions that were introduced into the Neubauer chamber for their observation under an optical microscope can be seen in Figure 2 (d and e, respectively, for the spleen and liver samples). In both samples, individual cells can be observed and the cell integrity was predominantly preserved without the presence of large cell aggregates. However, the images clearly show the larger size of the cells that are obtained from rat liver (Figure 2e) compared with the spleen cells (Figure 2d). Such results agree with the fact that hepatocytes (comprising about 75–80% of the total liver tissue cells [21]) have a mean size of 25–30 µm. Other cells that can be also present in the liver (e.g., Kupffer cells, etc.) in the range on 15–20 µm represent a minor cell population. On the other hand, spleen cells, mainly accounted for by T-cells (30–35% of the total) and B-cells (45–50% of the total), show sizes in the range of 5–7 µm and 7–8 µm, respectively [22]. 

Therefore, in view of these results, the optimized disaggregation protocol from tissues provides: (1) an adequate cell number concentration out of 0.5 g of tissue, (2) minimum cell debris, and (3) adequate cell morphology for subsequent single cell analysis. Further evaluation of the intracellular constitutive elemental analysis and the presence of unaltered cell surface receptors is necessary for further validation on the suitability of the procedure.

### 3.2. Analysis of Constitutive Elements by Single Cell ICP-MS Analysis

Trace elements present important structural and catalytic functions in the cell, so the accurate elemental analysis at the single cell level is of vital importance to understand biological processes such as metabolism, cell growth, or the development of diseases such as cancer [23]. In this regard, single cell ICP-MS has become a powerful tool to investigate the intracellular element content, providing additional information on cell heterogeneity, but it has been usually applied in in vitro studies using cell cultures [4,23,24]. In this work, the single cell suspensions that were obtained from disaggregated rat spleen and liver tissues were analyzed by SC-ICP-MS and, to our best knowledge, represents the first study using such an approach. 

**Figure 3:**
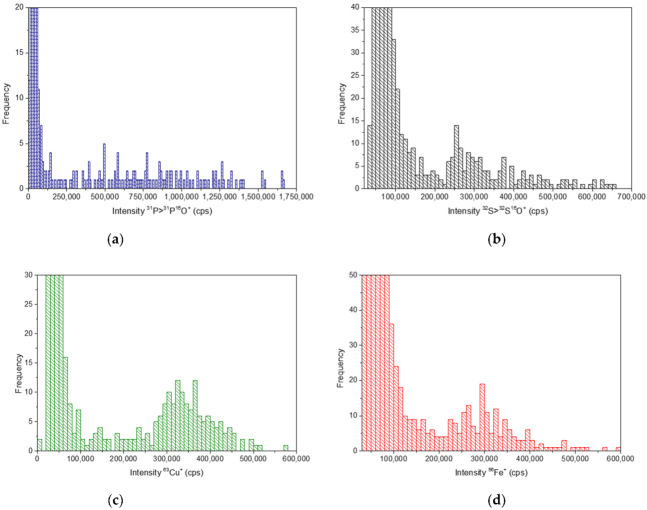
Histograms that show the signal distribution of spleen cells monitoring (**a**) ^31^P^+^ (as ^31^P^16^O^+^), (**b**) ^32^S^+^ (as ^32^S^16^O^+^), (**c**) ^63^Cu^+^ and (**d**) ^56^Fe^+^.

The fixed and washed cell suspensions were directly diluted in ultrapure water and were pumped at 10 µL min^−1^ to the high efficiency, pneumatic nebulization-based sample introduction system. Figure 3 shows the signals corresponding to P, S, Fe and Cu in spleen cells in the corresponding histograms [25]. Since the ICP-TQ-MS that was used in these studies allows the monitoring of just one isotope at a time, several runs had to be done in order to acquire these data, thus P and S were measured as oxides (^31^P^16^O^+^ and ^32^S^16^O^+^) and ^56^Fe^+^ and ^63^Cu^+^, on mass. 

Phosphorus is present in the phospholipids of the cell membrane, in the nucleic acids, as phosphate, and in a huge variety of biomolecules. Sulphur is mainly present in methionine and cysteine, which are two of the amino acids that constitute cell proteins. Iron and copper are essential elements whose metabolisms are closely related. Both are required as cofactors for the function of a wide variety of enzymes that are involved in relevant cellular processes such as oxygen transport, metabolism, or gene regulation. The results that were obtained (see Figure 3) showed relatively homogeneous cellular events for the cells that were obtained from the spleen tissues for all the analyzed elements and well separated from the background signal. Particularly remarkable were the signals that were obtained for ^63^Cu that showed an important homogeneity in the spleen samples. A lower number of events were detected in the liver samples, in agreement with the lower cell density, but showed intensity homogeneity as well. In all cases, the dilution that was carried out for the measurements was adequate to obtain an appropriate number of events (*n* > 100 in a 60 s run). It is also noteworthy the relatively high background that was observed for S, Cu, and Fe was probably ascribed to the disrupted cells. Since both the liver and spleen are rich tissues in metalloproteins containing S, Fe, and Cu, they should contribute significantly to the continuous signal that is observed that does not hamper the detection of the cell events.

**Figure 4:**
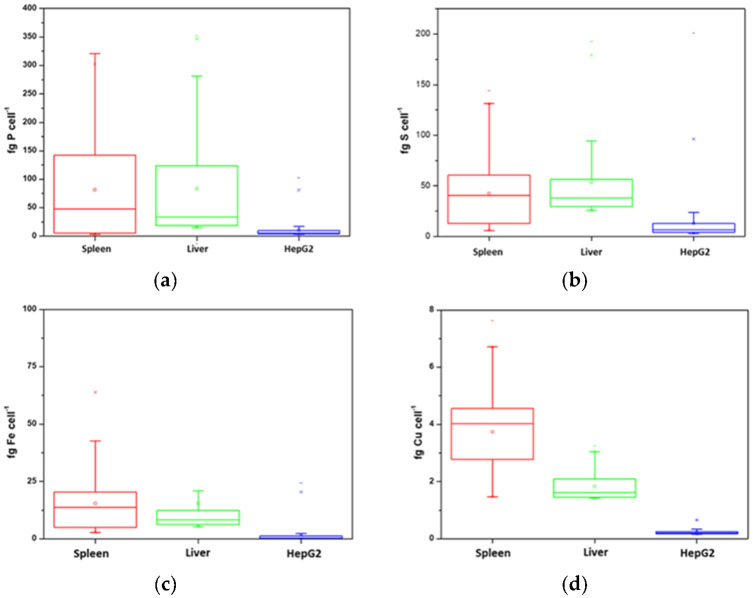
Boxplots showing the mass per cell of (**a**) phosphorus, (**b**) sulfur, (**c**) iron, and (**d**) copper in spleen (red), liver (green), and HepG2 (blue) cells.

According to the single cell ICP-MS principle, the intensity of the cellular events could be converted into the mass of each element per cell by using an external calibration with dissolved inorganic standards of the different elements that were measured using the same conditions as the cells and applying the previously established equation [19]. The equation implies the calculation of the transport efficiency of the liquid standards with the employed sample introduction system. In this case, it was determined by using 30 nm AuNPs and monitoring ^197^Au with 5 ms dwell time. The transport efficiency was calculated daily by comparing the number of detected events with the number of nanoparticles that were introduced into the plasma during the measurement acquisition time and was always about 65%. Using these calculations, the mass of each element per individual cell was obtained in the two investigated model samples (liver and spleen) and, also for comparison, in a cell culture of cancerous hepatic cells (HepG2). Figure 4 shows the quantitative results for all the analyzed elements as boxplots. 

In the first observation was a clear difference between the results that were obtained for the in vitro (cell cultures) and in vivo (animal models) experiments. A significantly lower elemental mass per cell of the four investigated elements was observed in the HepG2 cell model in comparison to the desegregated tissue cells. This is particularly remarkable in the case of Cu and points out the differences regarding cell cultures and cells that were extracted from real tissues. The cells that were obtained from the rat liver and spleen both showed similar contents of the four elements, although the heterogeneity between the individual cells is notably greater for the cells that were derived from spleen tissues. One explanation for this heterogeneity could be that while the liver is mainly constituted by hepatocytes (70–80%), spleen cells are more diverse comprising of macrophages, monocytes, and about one-fourth of the body’s lymphocytes coexisting in this organ [26]. A remarkable aspect is the high level of Fe that remains intracellularly in both types of cells from liver and from the spleen, in agreement with the fact that both tissues are accumulating high concentrations of this element which is mainly stored in the form of ferritin that can allocate up to 4500 atoms of iron. 

In any case, the obtained results further confirm the suitability of the disaggregation procedure to minimize cell disruption and to allow the monitoring of essential trace elements in individual cells, often hampered using imaging techniques (e.g., LA-ICP-MS).

### 3.3. Analysis of TfR1 in the Cell Surface by Single Cell ICP-MS

Iron, previously determined in all the samples, is an essential element for cellular development. In the human body, most stored iron is found in the liver, spleen, and bone marrow in the form of ferritin or hemosiderin [27]. The main entrance mechanism of Fe in cells occurs through its association with transferrin that is further bound to transferrin receptor 1 (TfR1) that is present on the cell surface. Additionally, in tumor cells, elevated TfR1 seems to be related to poorer outcome of patients [28]. Therefore, the TfR1 was evaluated as a cell biomarker model with the aim to address if the disaggregated cells maintain intact the cell surface receptors, which is one of the key aspects to be considered, particularly for further cell functional studies. 

**Figure 5:**
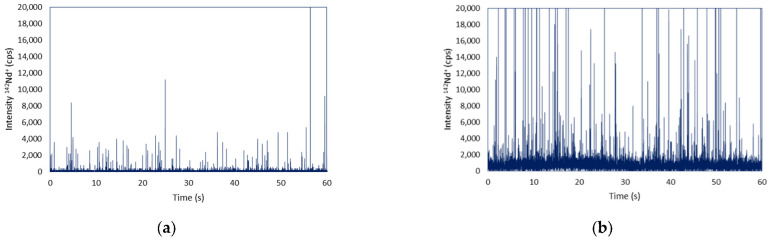
Time-resolved single cell ICP-MS analysis monitoring ^142^Nd^+^ in the (**a**) liver and (**b**) HepG2 cells after cell tagging with the Nd-conjugated antibody.

For this purpose, MAXPAR labelled antibodies were used for further SC-ICP-MS analysis. Following formaldehyde fixation of tissue-derived and HepG2 cells, all cell samples were tagged with the Nd conjugated antibody as previously described following the procedure that was optimized by Corte et al. [18]. The cells were washed again three times by centrifugation with TBS to remove the excess of the labelled antibody and/or residues of the labelling reagents. Figure 5 shows the ^142^Nd^+^ transient signals that were successfully obtained in the samples as a result of the TfR1 tagging with the antibody on the cell surface. It can be seen that the signals that were observed for the HepG2 are of significantly higher intensity than those from the disaggregated “healthy” organ. The intensity of these signals, as in the case of constitutive elements, was converted into the mass of Nd per cell by external calibration using Nd standards. In this case, considering the previously obtained stoichiometry of 21 Nd atoms per antibody [18], the Nd mass per individual cell could be converted into the number of TfR1 molecules per cell [29]. By using this factor, the quantitative results that were obtained for the three samples is shown in Table 1.

Although Figure 5a,b shows the same intensity for comparative purposes, it is difficult to see the lower intensity events (lower number of TfR1) of Figure 5a. However, after applying the 4σ criterion, the number of events that were obtained in both cases is shown in Table 1 and is relatively similar. According to these results, while the individual cells that were obtained from the rat liver and spleen present a similar number of TfR1 in their cell membrane, the HepG2 cells present a number of receptors about four- to five-fold higher. This observation agrees with the elevation of TfR1 in tumor cells since the HepG2 is a tumor cell line, while the other cells were obtained from healthy tissues. In addition, the results are in the same order of magnitude as those that were previously obtained using the same strategy for two cell models of breast cancer with different malignancy [18]. It is important to note here that with this methodology only the TfR1 that is present on cell membrane can be quantified and not the internalized forms of the receptor. 

These results further confirm the suitability of the implemented procedure of disaggregation of cells from tissue samples without significant alteration of the receptors that are present at the cell membrane. Altogether, the data that were obtained in this work reveal the promising capabilities of the proposed strategy to be applied in combination with mass cytometry but also to conduct SC-ICP-MS from small tissue sections with a minimum sample preparation for future cell studies.

## 4. Conclusions

In this work it is shown that single cell elemental analysis using inductively coupled plasma (ICP-MS) detection can be applied to more complex samples than isogenic cell cultures, opening a new world of opportunities. The development of a suitable sample preparation procedure for tissue disaggregation as shown here facilitates the use of SC-ICP-MS for validation of immunohistochemical techniques that are used for clinical diagnosis but also the cross-validation of LA-ICP-MS strategies. In this proof of concept study, the use of a one-step enzymatic cocktail has demonstrated to be adequate to isolate intact individual cells from tissues such as the liver or spleen with acceptable yields (about 20%). Disaggregated cells show adequate morphology and cell characteristics to perform further SC-ICP-MS of constitutive elements revealing a great homogeneity in the elemental content. However, when comparing “healthy” and cancerous cells, the elemental distribution of elements such as Cu and Fe seems to be very different which could serve as an advantage for cell discrimination. This is even more significant in the case of analyzing cell biomarkers such as TfR1 that shows different expression in cells from tumor origin and non-tumor cells. These results altogether confirm the great potential of the SC-ICP-MS approach using high efficiency pneumatic nebulization-based sample introduction systems for future clinical and biomedical applications.

## Figures and Tables

**Table 1:** The average Nd mass per cell and the average number of TfR1 per cell that were obtained by single cell ICP-MS after cell tagging with the labelled antibody.

	Spleen	Liver	HepG2
Nd mass per cell (fg)	0.088 ± 0.001	0.089 ± 0.001	0.231 ± 0.09
TfR1 per cell	(1.86 ± 0.15) × 10^4^	(1.86 ± 0.17) × 10^4^	(4.97 ± 1.96) × 10^4^
Number of events	279	195	202

## Data Availability

Not applicable.

## Supplementary Materials

The following supporting information can be downloaded at: https://www.mdpi.com/article/10.3390/nano13010012/s1, Table S1: iCAP TQ ICP-MS operating conditions in single cell mode. Figure S1: Image of the sample introduction system that was used. Taken from Ref. [30]; Figure S2: Time-resolved single cell ICP-MS analysis of spleen cell monitoring (A) ^31^P^+^ (as ^31^P^16^O^+^), (B) ^32^S^+^ (as ^32^S^16^O^+^), (C) ^63^Cu^+^, and (D) ^56^Fe^+^.
