# Supplementary material for: Capabilities of Single Cell ICP-MS for the Analysis of Cell Suspensions from Solid Tissues
Source: Nanomaterials (Basel). 2022 Dec 20;13(1):12. doi: 10.3390/nano13010012 (PMC9823448; doi:10.3390/nano13010012)
Supplement: Supplementary file 1 [file nanomaterials-13-00012-s001.zip › nanomaterials-2070418-supplementary.pdf]

Table S1. iCAP TQ ICP-MS operating conditions in single cell mode.

Instrument	iCAP TQ ICP-MS			
RF Power [W]	1550			
Coolant gas flow [L min^{-1}]	14.0			
Auxiliary gas flow [L min^{-1}]	0.8			
Nebulizer gas flow [L min^{-1}]	0.52			
Sheath gas flow [L min-1]	0.65			
Measurement mode	TQ-O_2	SQ-He	SQ-H_2	SQ
Analyte isotope	^{31}P, ^{32}S	^{63}Cu	^{56}Fe	^{142}Nd, ^{197} Au
Cell gas flow	0.32	5.9	7.6	-
Q1 masses [u]	31, 32	-	-	-
Q3 masses [u]	47, 48	63	56	142, 197
Q1 bias [V]	0			
Qcell bias [V]	-5,94			
Q3 bias [V]	-12			
Dwell time [ms]	5			
Sample flow rate [mL min^{-1}]	0.01			

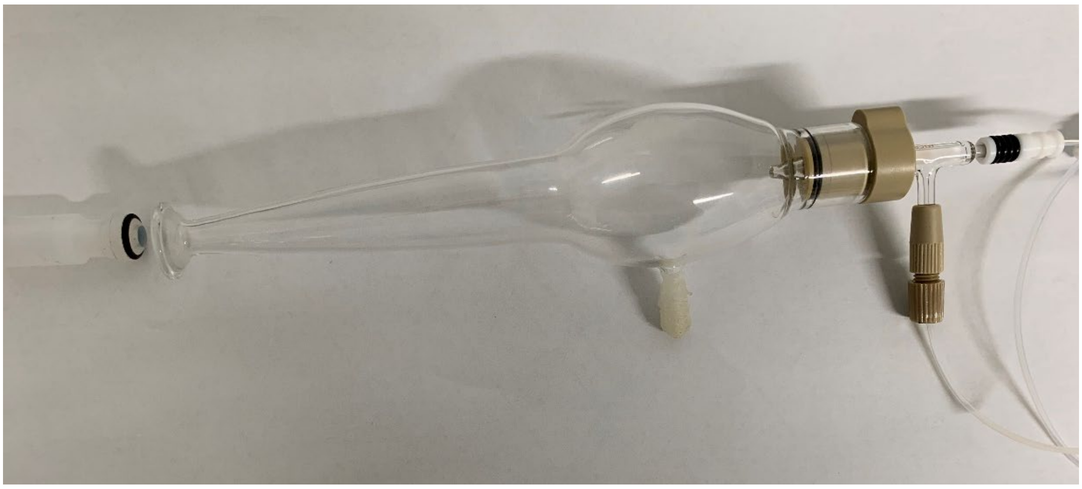

Figure S1. Image of the components of the sample introduction system used. Details can be seen in reference [30].

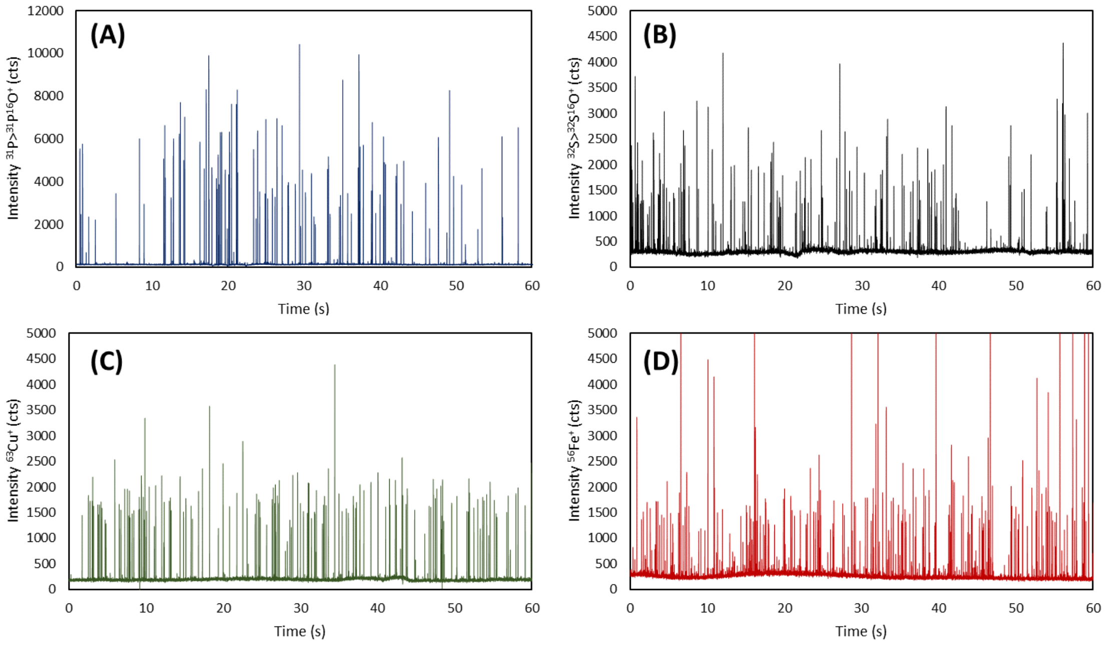

Figure S2. Time-resolved single cell ICP-MS analysis of spleen cells monitoring (A) $^{31}P^{+}$ (as $^{31}P^{16}O^{+}$), (B) $^{32}S^{+}$ (as $^{32}S^{16}O^{+}$), (C) $^{63}Cu^{+}$ and (D) $^{56}Fe^{+}$.
